# Commentary: Unlearning implicit social biases during sleep

**DOI:** 10.3389/fpsyg.2015.01428

**Published:** 2015-09-23

**Authors:** Balazs Aczel, Bence Palfi, Barnabas Szaszi, Aba Szollosi, Zoltan Dienes

**Affiliations:** ^1^Institute of Psychology, Eotvos Lorand UniversityBudapest, Hungary; ^2^School of Psychology, University of SussexBrighton, UK

**Keywords:** social biases, IAT, sleep, memory reactivation, system-level consolidation process

Without question, reducing social biases is one of the main aims and challenges of modern societies. Implicit biases have been shown to be robust findings in social psychology, popularly measured by the IAT. Recently, Hu et al. (2015) developed a training to counter these biases relying on the system-level consolidation process, in which repeated reactivation of information during sleep is aimed to improve post-sleep memory performance. As a baseline test, participants (*N* = 40) completed two social bias IATs where the face-word assignments were either consistent or inconsistent with the typical bias. In their counter-bias training counter-gender and counter-racial bias trials were paired with different sound cues. All participants took part in a nap session where one of the sound cues was repeatedly presented as they entered slow-wave sleep (SWS). 10 min and 1 week after their nap, the participants completed more IATs to test the endurance of the training for cued and uncued biases. Based on their results, Hu et al. (2015) claimed that (1) implicit social biases were reduced by the counter-stereotypic training; (2) this bias reduction was fortified by memory reactivation during sleep; and (3) the long-term effect of the training is sleep-dependent, as the reduction remained only for the social bias being cued during sleep.

The most important promise of this research is an application of this sleep-training in a potential bias-reducing intervention. The crucial evidence here would be the maintained benefit that the training can produce between the baseline and a later occasion. The relevant analysis, therefore, is where Hu et al. (2015) compared the change between the baseline and the delayed bias assessment, differentiated by the performance on the cued and uncued biases. Specifically, Hu et al. (2015) conducted a Two-Way within-subjects ANOVA (Sleep training × Time) on IAT scores as dependent variable and they compared the baseline time point with the delayed time point. Neither the interaction (Sleep training × Time), *F*_(1, 37)_ = 0.471, *P* = 0.497, nor the main effect of Time were significant, *F*_(1, 37)_ = 3.095, *P* = 0.087 (only the interaction was reported). Although the interaction was non-significant, the authors conducted uncorrected *post-hoc t*-tests between the two times finding that the cued biases got significantly weaker, *P* = 0.034, while the uncued biases did not, *P* = 0.603. This simple declaration of the significant and non-significant results reflects a common statistical fallacy by suggesting that the two conditions had different effect (Gelman and Stern, 2006; Nieuwenhuis et al., 2011), whereas the interaction was not significant. Traditionally, the non-significant interaction should disallow the subsequent *t*-tests and non-significant change could not be interpreted as no change in uncued biases. To ascertain whether there is evidence for a null hypothesis, or rather just no evidence either way, we conducted a Bayesian analysis on the original data. Bayes Factor (B) takes into account the effect size obtained, its standard error, and the rough effect size predicted in an optimum way to produce a measure of strength of evidence for the alternative hypothesis (H1) rather than the null hypothesis (H0). By convention (Jeffreys, 1961; Dienes, 2014), a B more than 3 is substantial evidence for H1; less than 1/3 is substantial evidence for H0; and in between 1/3 and 3 is not much evidence either way. A *p*-value by itself does not allow a non-significant result to be interpreted. A Bayes factor allows statements of strength of evidence by modeling the predictions of H1, i.e., taking into account the rough size of effect predicted. Dienes (2014) recommends modeling H1 with a half-normal for a theory making a directional prediction.

Taking a half-normal distribution with a standard deviation of the half of the average range measured in the Baseline condition (*SD* = 0.28) to model the predictions of H1 (see Figure 1), yields *B* = 0.72 for the interaction and *B* = 1.56 for the main effect of time. Both Bayes Factors indicate that we don't have evidence for the efficacy of the training procedure in the long run, because the data are insensitive.

**Figure 1:**
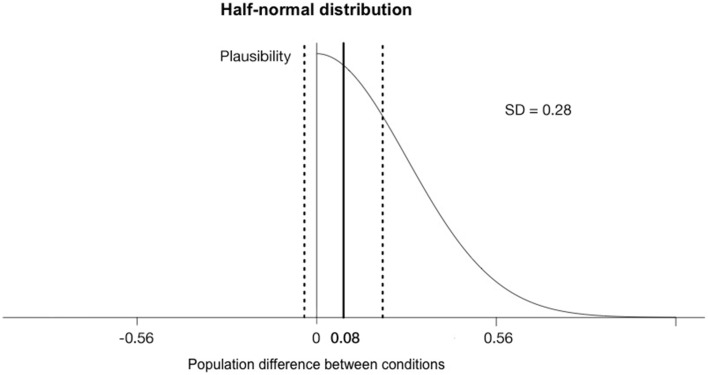
**The half-normal distribution model of the predictions of H1 with mode of zero and standard deviation of half of the Baseline value (*SD* = 0.28)**. The plausibility of the expected effect size decreases with the extent of the effect size. The bold vertical line shows the mean difference (0.08) of the interaction (Sleep training × Time) and the dashed lines indicate the standard error of the difference (0.12).

Hu et al.'s study showed convincing evidence that their sleep-training can considerably decrease social biases as measured by the IAT immediately after the participants' nap. We also agree with the authors that “maintaining the benefits of training is crucial for the ultimate usefulness of potential bias-reducing interventions” (p. 1014) as it would serve great public benefit. Nevertheless, the results of the current experiment are inconclusive as to whether memory reactivation during sleep enhances counter-stereotype training or if bias reduction is sleep-dependent. Bayes Factor calculation could help the authors finalize their analysis by additional data collection. Bayes Factor as a measure of strength of evidence is not sensitive to optional stopping (Rouder, 2014), it enables researchers to collect data until discrimination is reached (*B* > 3 or < 1/3). Therefore, the present study could be concluded when the study meets the level of sensitivity sufficient to evaluate the model, but until then we have no evidence that sleep-training can be used as an intervention to reduce social and gender biases in real life.

## Conflict of interest statement

The authors declare that the research was conducted in the absence of any commercial or financial relationships that could be construed as a potential conflict of interest.
